# Noncanonical Signaling Network of Nogo-A: A New Perspective on Multi-target Regulation, Neuroplasticity, and Disease Intervention Beyond NgR

**DOI:** 10.1007/s10571-026-01787-7

**Published:** 2026-08-07

**Authors:** Tao Yang, Yongxiang Yang, Jingmin Cheng, Kexia Fan, Yuan Ma, Dongbo Zou, Haifeng Shu, Sixun Yu

**Affiliations:** 1 Department of Neurosurgery, The General Hospital of Western Theater Command, Chengdu, 610038 Sichuan China; 2 Southwest Jiaotong University, Chengdu, 610054 Sichuan China

**Keywords:** Nogo-A, Synaptic plasticity, Alzheimer’s disease, Neuroinflammation, Microglia

## Abstract

The membrane protein Nogo-A, initially identified for its inhibitory role in axonal regeneration through the Nogo receptor (NgR), is now recognized as a central hub in a complex noncanonical signaling network. Recent evidence indicates that Nogo-A engages diverse receptors and intracellular pathways beyond NgR to modulate neuroplasticity, synaptic stability, and glial function. This multi-target regulatory capacity characterizes Nogo-A as a critical determinant of neural repair and degeneration, with implications for conditions such as spinal cord injury, multiple sclerosis, and Alzheimer’s disease. Noncanonical Nogo-A signaling intersects with growth factor cascades, inflammatory responses, and cytoskeletal dynamics, offering novel therapeutic targets for neural repair. This review synthesizes current knowledge on alternative Nogo-A signaling mechanisms and highlights their potential to redefine strategies for neuroregeneration and disease intervention.

**Graphical Abstract:**

**Figure:**
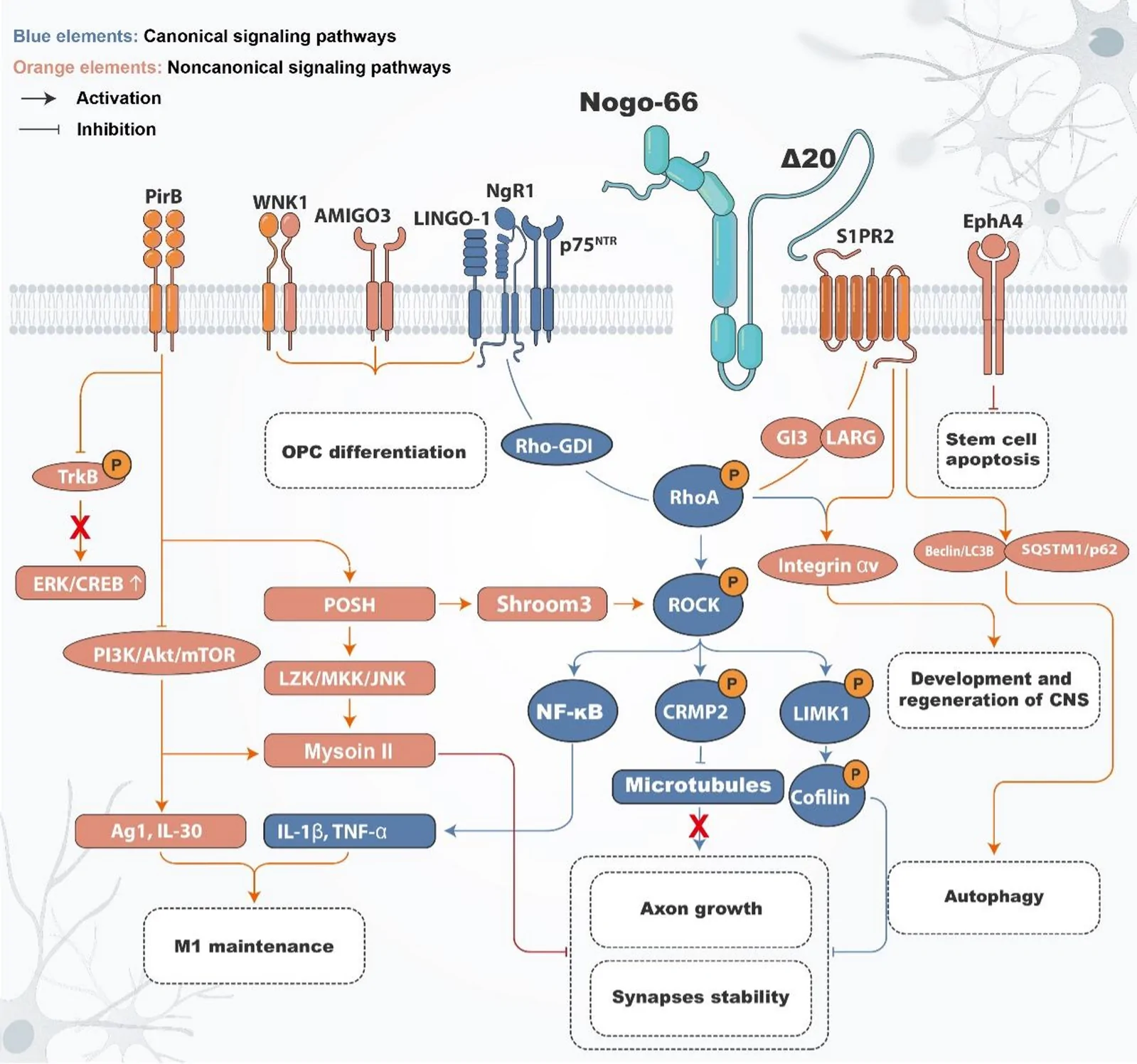

Noncanonical Nogo-A Signaling Network. This graphical abstract summarizes the Nogo-A signaling network. It illustrates how Nogo-A engages diverse receptors (e.g., PirB, S1PR2) beyond the classical NgR to activate divergent pathways, modulating neuroplasticity and offering novel therapeutic targets for neural repair

## Introduction

Nogo-A, has long been characterized as a myelin-associated inhibitory factor that restricts axonal regeneration and neural plasticity through the Nogo-66 receptor 1 (NgR1) and its co-receptors, including p75^NTR^ and LINGO-1 (Abdesselem et al. 2009; Mohammed et al. 2020; Iobbi et al. 2017; Kellner et al. 2016). However, accumulating research suggests that Nogo-A functions are not confined to the classical NgR1 pathway. Various non-classical receptors and interaction partners play significant roles in regulating synaptic function, neural network remodeling, and neurodevelopmental disorders. Recent studies have shown that Nogo-A engages in molecular crosstalk with integrins, extracellular matrix components, and growth factor receptors such as TrkB, thereby influencing synaptic plasticity and circuit remodeling (Johnstone and Mobley 2020; Seiler et al. 2016; Farrer and Kartje 2025). The expression and function of receptors or co-receptors like PirB, LINGO-1, and AMIGO3 vary across brain regions, demonstrating region-specific multi-level regulatory characteristics (Fang et al. 2025; Farrer and Kartje 2018; Kalafatakis et al. 2023; Lang et al. 2017; Park et al. 2021; Pavon et al. 2024).

In this review, “noncanonical Nogo-A signaling” is defined as the regulatory network involving receptors and intracellular pathways beyond the classical NgR1/p75/LINGO-1 complex. This includes interactions with sphingosine-1-phosphate receptor 2 (S1PR2), paired immunoglobulin-like receptor B (PirB), EphA4, and PlexinA2, as well as the roles of Nogo-A in non-neuronal contexts such as vascular remodeling and metabolic signaling. Unlike the classical pathway, which primarily acts as a “brake” on structural growth, noncanonical signaling functions as a dynamic integrator of environmental cues, coordinating responses across different cell types and pathological stages. In this review, we operationally define noncanonical Nogo-A signaling into two distinct levels: direct receptor-mediated pathways and the downstream, secondary systemic effects.

## Diversity and Specific Functions of Nogo-A Receptors and Co-receptors

**Fig. 1:**
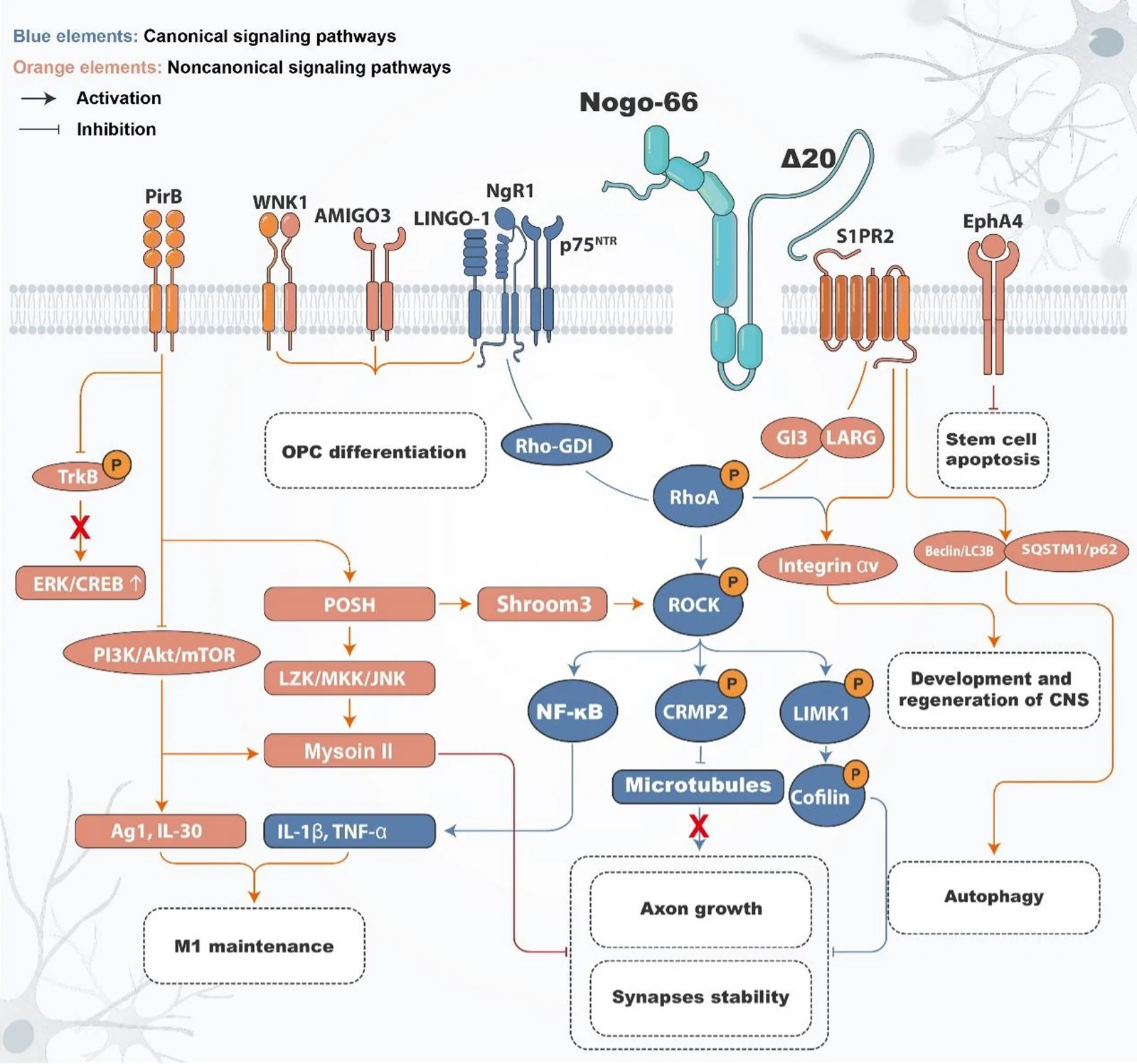
Schematic representation of Nogo-A-associated signal transduction mechanisms. The diagram illustrates the interaction between Nogo-A domains (Nogo-66 and Δ20) and their respective membrane receptors. (Note: Please ensure your colors match this description) Blue elements denote the canonical NgR1-mediated signaling complex (NgR1, p75^NTR^, LINGO-1) and its co-receptors, which predominantly activate the RhoA/ROCK cascade and NF-κB to regulate axon growth, synaptic stability, and microglial polarization. Orange elements represent noncanonical signaling pathways. PirB suppresses the PI3K/Akt/mTOR axis and TrkB phosphorylation, while activating the POSH/LZK/MKK/JNK cascade to regulate M1 microglia maintenance and OPC differentiation. The Nogo-A Δ20 domain interacts with S1PR2 to initiate autophagy and integrin-mediated RhoA activation, and with EphA4 to modulate stem cell apoptosis

Nogo-A interacts with multiple membrane receptors and co-receptors to form a complex signaling network that is pivotal for neuroplasticity and disease progression. Receptors such as NgR1, PirB, and LINGO-1 recruit downstream signaling molecules through distinct domains, including the leucine-rich repeat (LRR) motif of NgR1 and the immunoreceptor tyrosine-based inhibition motif (ITIM) of PirB. These interactions dynamically modulate pathways such as RhoA/ROCK and PI3K/Akt/mTOR. Figure 1 illustrates these classical and non-classical signaling pathways. The spatiotemporal-specific coupling of Nogo receptor-pathway interactions underpins the heterogeneity of the Nogo-A signaling network.

### Classical NgR1 Signaling Mediated by the Core Receptor Complex

NgR1 is one of the primary Nogo-66 receptor proteins identified. It is extensively expressed on the cell membrane and participates in various biological processes. NgR1 forms a ternary complex with p75^NTR^ and LINGO-1, which activates the RhoA/ROCK signaling pathway to inhibit axon outgrowth and synaptic remodeling. The underlying molecular mechanism suggests that the NgR1 complex influences neuronal morphology and modulates synaptic function and plasticity, thereby determining the stability of neural circuits (Li et al. 2021; Lee et al. 2008; Yang et al. 2017; Karlsson et al. 2016). Research indicates that the NgR1 complex maintains synaptic stability by regulating F-actin dynamics in dendritic spines and restricting AMPAR insertion. Conversely, the absence or blockade of NgR1 enhances the stability of NK cell-target contacts and regulates F-actin dynamics during immunological synapse formation. Collectively, these findings suggest that NgR1 serves as a core regulator of synaptic plasticity balance through precise control of the cytoskeleton. This regulation safeguards neural network stability while limiting excessive synaptic fluctuations to ensure reliable information transmission (Lee et al. 2008; Oh et al. 2023). In the Schaffer-CA1 pathway, components of the NgR1 complex, including p75^NTR^ and LINGO-1, exhibit distinct functional tendencies, although their ultimate regulatory roles on long-term potentiation (LTP) and long-term depression (LTD) are highly context-dependent (Iobbi et al. 2017). P75^NTR^ is not essential for LINGO-1-mediated NgR1 potentiation. This discovery refines the understanding of NgR1 in synaptic plasticity (Raiker et al. 2010; Lee et al. 2008; Iobbi et al. 2017). Recent studies also show that NgR1 transcriptionally regulates the WNK1 gene to mediate cell migration, proliferation, and axon extension. Furthermore, WNK1 interacts with LINGO-1 through its kinase domain to mediate the inhibitory effect of Nogo-66 on oligodendrocyte precursor cell (OPC) differentiation (Zhang et al. 2015). This regulatory mechanism operates independently of the classical Nogo-A/NgR1 ligand-binding pathway (Yang et al. 2017; Andrews et al. 2018).

### PirB and its Downstream PI3K/Akt/mTOR Signaling Pathway

Paired immunoglobulin-like receptor B (PirB) also exerts critical regulatory functions in the central nervous system. As a high-affinity alternative receptor for Nogo-66, PirB suppresses neuronal growth and plasticity through several molecular pathways (Atwal et al. 2008; Kurihara et al. 2020; Yang et al. 2021). Specifically, PirB function is mediated by the inhibition of the PI3K/Akt/mTOR signaling pathway. This axis serves as a core signaling hub that regulates growth and metabolism, and its suppression directly impedes axon elongation and neuronal branching (Bi and Quan 2018; Zhao et al. 2017). Experiments in neonatal mouse cortical neuron cultures demonstrated that PirB expression is significantly upregulated following Nogo-66 treatment, accompanied by altered molecular reactions associated with axonal growth (Fig. 1). Reduced PI3K/Akt/mTOR activity leads to decreased phosphorylation of downstream effectors such as myosin IIA and cofilin, which are critical for maintaining cytoskeletal integrity. Consequently, these changes inhibit neurite outgrowth and reduce neuronal branching. These inhibitory effects are partially reversed by anti-PirB antibodies or specific PI3K/mTOR inhibitors (Bi and Quan 2018; Zhao et al. 2017; Hixson et al. 2019). PirB is also expressed in primary retinal ganglion cells (RGCs). Adenovirus-mediated knockdown of PirB enhances RGC neurite regeneration and activates the JAK/Stat3 signaling pathway in Müller glial cells while upregulating ciliary neurotrophic factor levels (Yuan et al. 2020). These findings indicate that the PirB/PI3K/Akt/mTOR signaling axis is critical for neural development and wound healing. In a physiological state, this pathway maintains neural homeostasis by inhibiting excessive growth or abnormal synaptic connections. In cases of nerve injury or neurodegeneration, the suppression of this mechanism may present a significant barrier to regeneration. Parallel to PI3K/Akt/mTOR signaling, PirB can also inhibit cofilin dephosphorylation via the RhoA/ROCK pathway, leading to cytoskeletal contraction and growth cone collapse (Li et al. 2023). Furthermore, PirB regulates neural stem cell behavior through the Shp1/2 signaling pathway (Ramasamy et al. 2014), making it a promising target for regenerative medicine.

### LINGO-1, p75^NTR^, and the ROCK2-Cofilin Pathway

LINGO-1, as a core receptor of NgR1, plays a crucial role in Nogo-66-mediated suppression of LTP. Depletion experiments showed that inhibiting LINGO-1 enhances LTP levels in the Schaffer-collateral CA1 pathway, while p75^NTR^ predominantly mediates LTD (Iobbi et al. 2017).

p75^NTR^ is a transmembrane glycoprotein of the tumor necrosis factor receptor (TNFR) superfamily. It binds to various ligands, including neurotrophic factors such as NGF, BDNF, NT-3, and NT-4, as well as pro-neurotrophic factors and amyloid beta-peptides. Through interactions with co-receptors and intracellular adaptor proteins, p75^NTR^ activates cascades crucial for neuronal growth, differentiation, and survival (Yang et al. 2022). Beyond its role in NgR1-dependent LTD regulation, p75^NTR^ interacts with the Fas receptor (Shanks et al. 2024), contributing to synaptic plasticity and degeneration via the Fas/CD95 ligand system, primarily by activating RhoA signaling. In Alzheimer’s disease, p75^NTR^ expression is upregulated and interacts with amyloid beta-peptides to induce apoptosis and inflammatory responses (Eggert et al. 2022; Shanks et al. 2024). In Parkinson’s disease, p75^NTR^ may contribute to the degeneration of dopaminergic neurons (Ali et al. 2024).

Mechanistically, the Nogo-66/NgR1/LINGO-1 complex regulates dendritic spine F-actin dynamics and modulates synaptic strength through the ROCK2-Cofilin pathway (Iobbi et al. 2017). In neural stem cell differentiation and spinal cord injury models, LINGO-1 influences axon regeneration and nerve survival by regulating ERK and CREB signaling. MicroRNAs such as miR-615 and miR-182-5p have been confirmed to downregulate LINGO-1 expression, promoting neural differentiation and providing novel insights for gene therapy (Atwal et al. 2008; Kurihara et al. 2020).

### The Role of AMIGO3 in the Post-epileptic State and Developmental Abnormalities

As a homologue of LINGO‑1, AMIGO3 has recently been identified as an NgR1 co-receptor in certain pathological conditions, functioning instead of LINGO‑1. Studies have demonstrated that the expression levels of both AMIGO3 and LINGO‑1 in the hippocampus of mice following childhood epilepsy progressively increase over time. Myelin damage and axonal growth restriction are mediated via the ROCK/RhoA signaling pathway. Inhibition of AMIGO3 expression not only alleviates myelin structural damage but also enhances the repair capacity of neural circuits (Li et al. 2021). These findings indicate that AMIGO3 plays a critical role during various developmental stages and disease progression, representing a promising regulatory target for neural remodeling following multiple sclerosis and inflammatory injuries.

### Novel Receptors: PlexinA2/CRMP2, EphA4, S1PR2

PlexinA2, a neuroguide receptor, has recently been identified as a critical coreceptor of NgR1. Upon Nogo-A stimulation, the interaction between NgR1 and CRMP2 is mediated by PlexinA2 to form the NgR1/PlexinA2/CRMP2 ternary complex, thereby activating RhoA signaling and inhibiting cortical axon regeneration and functional recovery (Sekine et al. 2019). Moreover, NgR1 relies on PlexinA2 in neurons for regulating axon guidance during chick spinal cord development and in developing dI1 neurons. Wang et al. also demonstrated that Nogo-A Δ20 can directly bind to EphA4, inhibiting neural stem cell apoptosis through balanced p38 and JNK MAPK signaling, thus revealing a novel molecular interaction between Nogo-A and EphA4 (Wang et al. 2021a). S1PR2 (sphingosine-1-phosphate receptor 2) has been shown to form a complex that negatively regulates angiogenesis with Nogo-A, and its activation enhances vascular endothelial autophagy, suppresses regeneration, and impairs nerve repair following midbrain infarction (Tang et al. 2020; Dong et al. 2025). Nogo-A influences nervous system development and regeneration by binding to multiple distinct receptors (such as S1PR2, integrin αv, and HSPGs), which independently activate the downstream RhoA/ROCK signaling pathway. (Pradhan and Das 2021). The structural features, binding ligands, downstream pathways, and pathological roles of these major canonical and noncanonical Nogo-A receptors are systematically summarized in Table 1.

**Table 1:** Functional comparison of major Nogo-A receptors

Receptor	Structural features	Binding ligands	Downstream pathways	Physiological functions	Pathological associations	References
NgR1	LRR domain + GPI anchor	Nogo-66, MAG, OMgp	RhoA/ROCK, WNK1	Synaptic stability maintenance; Growth cone collaps, neurite outgrowth was inhibited; Regulating Macrophages	Alzheimer’s disease (Aβ deposition), spinal cord injury (axonal inhibition); vascular endothelial autophagy	Gopalakrishna et al. (2020); Xu et al. (2020); Pavon et al. (2023); Soto et al. (2022); Zhang et al. (2021, 2022); Iobbi et al. (2017)
PirB	Ig-like domains + ITIM motifs	Nogo-66, myelin inhibitors	PI3K/Akt/mTOR, Shp1/2, POSH/Mysoin II	Neurite branching restriction, neural stem cell survival and proliferation	Multiple sclerosis (impaired remyelination)	Ramasamy et al. (2014); Liu et al. (2019); Bi and Quan (2018); Zhao et al. (2017); van der Touw et al. (2018); Dickson et al. (2010)
LINGO-1	LRRNT domain + transmembrane region	NgR1 complex	ERK/CREB, ROCK2-Cofilin	LTP suppression, Remyelination and Regeneration, OPC differentiation	Multiple sclerosis, Neuromyelitis optica (biomarker)	Iobbi et al. (2017); Kalafatakis et al. (2023); Zhao et al. (2007); Satoh et al. (2007)
S1PR2	Sphingosine-1-phosphate receptor	Nogo-A, S1P	RhoA/ROCK, autophagy	Angiogenesis regulation, repressing synaptic plasticity	Stroke (vascular endothelial autophagy)	Xiao et al. (2022); Tang et al. (2020); Kempf et al. (2014)

*LRR* leucine-rich repeat, *ITIM* immunoreceptor tyrosine-based inhibitory motif, *LRRNT* leucine-rich repeat N-terminal domain, *S1P* sphingosine-1-phosphate, *OPC* oligodendrocyte precursor cells

## Logic of Signal Integration: Noncanonical Receptors in Extended Crosstalk Networks

A critical challenge in Nogo-A biology is understanding how cells integrate signals when multiple receptors are co-expressed. The noncanonical network operates as an integrated logic circuit.

### The Crosstalk Between Nogo-A Receptors

The integration of Nogo-A signals often involves the convergence of distinct receptors on shared downstream effectors. For example, both S1PR2 and NgR1 activate the RhoA/ROCK pathway, though through different proposed pathways. S1PR2 activates RhoA via G12/13 and RhoGEFs like LARG, while NgR1 utilizes co-receptors like p75^NTR^ (Kempf et al. 2014). When both are present, they are hypothesized to synergistically amplify the inhibitory signal, establishing a robust dual-pathway suppression of axonal growth (Kempf et al. 2014; Schwab and Strittmatter 2014).

Conversely, competitive binding for Nogo-A domains can modulate the signaling output. The Nogo-A Δ20 domain specifically binds to S1PR2, while the Nogo-66 domain binds to NgR1 and PirB. The relative availability of these domains, regulated by proteolytic cleavage or protein folding, determines receptor prioritization (Fournier et al. 2001).

The most sophisticated crosstalk occurs between S1PR2/NgR1 (RhoA-driven) and PirB (SHP-1/2-driven) signaling. While RhoA primarily drives physical changes in the cytoskeleton, PirB recruits the phosphatases SHP-1 and SHP-2 via its ITIM motifs (Atwal et al. 2008). These phosphatases can dephosphorylate and inhibit pro-growth signaling hubs such as the PI3K/Akt/mTOR axis and TrkB receptors (Zhao et al. 2017). This integration establishes a dual-layered inhibitory logic. The first layer consists of structural inhibition mediated by RhoA, inducing rapid cytoskeletal retraction for immediate physical constraint. The second layer involves metabolic and growth arrest executed by SHP1/2, suppressing biosynthetic pathways for sustained arrest. This coordinated design ensures robust signal enforcement, as cells remain in a quiescent state even if the structural brake is bypassed (Fournier et al. 2001).

When NgR1, PirB, and S1PR2 are co-expressed on the cell membrane, the final direction of downstream signaling is not a simple linear summation, but a highly coordinated hierarchical integration. First, this hierarchy is dictated by binding affinity differentials, wherein the canonical NgR1 complex serves as the primary high-affinity binding platform for the Nogo-66 domain (Fournier et al. 2001), while PirB acts as a functional alternative or co-receptor to fine-tune structural plasticity thresholds (Atwal et al. 2008; Kurihara et al. 2020). Second, spatial compartmentalization exerts a decisive regulatory influence; the NgR1 complex is preferentially clustered within specific membrane microdomains, such as lipid rafts, ensuring its localized activation at targeted synaptic sites to restrict structural dynamics and preserve neural circuit stability (Lee et al. 2008; Iobbi et al. 2017). Finally, the pathological microenvironment can dynamically rearrange this signaling hierarchy. For example, during the acute ischemic phase following stroke or Central Nervous System (CNS) injury, local microenvironmental cues shift the balance toward the preferential and rapid activation of localized S1PR2 pathways, inducing immediate RhoA-mediated physical constraints over sustained metabolic arrest (Kempf et al. 2014; Tang et al. 2020). This context-dependent flexibility allows the noncanonical Nogo-A signaling network to transition seamlessly between protective stabilization and pathological inhibition depending on the disease stage.

### Interaction of Nogo-A with Integrins and Extracellular Matrix Components

Molecular interplay between Nogo-A, the integrin family, and extracellular matrix (ECM) components has also gained attention. The canonical interaction mechanism involving integrins and receptor tyrosine kinases (RTKs) in regulating cell adhesion offers theoretical support for Nogo-A-integrin interactions within GPCR-RTK heterocomplexes (Di Liberto et al. 2019; Hu and Strittmatter 2008; Park et al. 2021). In multiple sclerosis models, Nogo-A modulation of astrocyte morphology may involve ECM remodeling, which restricts synapse formation (Espírito-Santo et al. 2021). This non-canonical binding mode suggests a novel role for Nogo-A in cell adhesion and matrix regulation.

### Cross-Talk with the Growth Factor Receptor TrkB

When investigating the regulatory mechanisms of neurotrophic factors, the spatially specific regulation of TrkB signaling microdomains has garnered significant scholarly attention. The local signaling network mediated by this receptor exerts a unique regulatory influence on synaptic remodeling and axon branching (Johnstone and Mobley 2020). However, this regulation may be disrupted by interactions with Nogo-A. Studies indicate that in cerebral ischemia models, Nogo-A forms a complex with PirB to suppress TrkB phosphorylation, reducing ERK/CREB signaling activity (Liu et al. 2019). This inhibitory effect is not entirely unidirectional, as activation of the TrkB pathway following intervention with specific inhibitors can overcome the Nogo-A/PirB blockade. Overexpression of Nogo-A also enhances its physical interaction with TrkA and interferes with normal Erk1/2 transmission by shortening receptor phosphorylation (Farrer and Kartje 2018, 2025).

This cross-inhibitory effect suggests that neurotrophin signaling exists in a dynamic balance with inhibitory pathways. Accumulating evidence points toward a cross-inhibitory hypothesis, where Nogo-A may compete for receptor dimerization interfaces, thereby indirectly modulating growth cone guidance and neuronal survival (Farrer and Kartje 2025; Kilic et al. 2010; Wang et al. 2021b).

### Heterologous Complexes with RTKs and GPCRS

The phenomenon of GPCR/RTK heterodimerization is not novel. As early as 2008, the co-activation mechanism of CB1 receptors and TrkB receptors refined our understanding of therapeutic targets for depression. However, blocking this receptor complex can reverse stress-induced synaptic loss (Lutz 2020; Di Liberto et al. 2019). Similarly, Nogo-A regulates the PI3K pathway by bridging GPCRs (such as S1PR2) with RTKs (such as TrkB) through NgR1 to form ternary complexes. For instance, in astrocytes, the Nogo-A/NgR1/S1PR2 complex activates PI3K in a pulsatile manner, whereas TrkB activation alone induces a sustained signal (Di Liberto et al. 2019). This dynamic allosteric property may elucidate the dual role of Nogo-A signaling in neuroprotection versus inflammation suppression.

### Metabolic and Mechano-Chemical Integration of Downstream Cascades

Subcellular compartmentalization and spatial microdomains dictate how conflicting extracellular cues are resolved when multiple noncanonical Nogo-A receptors are co-expressed. A-kinase anchoring proteins (AKAPs) and mammalian target of rapamycin (mTOR) complexes establish localized signaling outposts within distinct neuronal compartments to regulate spatial homeostasis (Altas et al. 2022; Sadeghian et al. 2022). In mammalian cortical neurons, local growth cone translation is precisely managed by the mTORC1 pathway via effectors such as 4E-BP1 (Altas et al. 2022). Noncanonical Nogo-A signaling directly intersects with this metabolic hub by restricting the subcellular localization and activation of mTORC1, thereby enforcing a state of local translational quiescence that halts growth cone dynamics and axonal regeneration (Altas et al. 2022). This localized spatial gating demonstrates that Nogo-A does not merely act as a passive physical barrier, but actively orchestrates the spatial rearrangement and metabolic capacity of the local protein synthesis machinery.

This localized metabolic control operates in tandem with broader downstream homeostatic and mechano-chemical networks, including the JAK/STAT and Hippo-YAP/TAZ pathways, to shape the macro-environment of neural repair. While the JAK/STAT system modulates long-term transcriptional regulation under continuous trophic exposure, its activation interacts with noncanonical Nogo-A signals to determine cellular phenotypic shifts (Hixson et al. 2019; Sun et al. 2023). Concurrently, the Hippo pathway effectors YAP and TAZ sense and translate mechanical cues from extracellular matrix stiffness into adaptive transcriptional programs (Dupont et al. 2011; Ajongbolo and Langhans 2025). Because Nogo-A induces robust actin cytoskeleton contraction via the RhoA/ROCK axis, it directly modulates the intracellular mechanical tension that regulates YAP/TAZ stability and nuclear translocation (Kempf et al. 2014; Schwab and Strittmatter 2014). This mechano-chemical coupling establishes a critical feedback loop where Nogo-A-driven physical tension alters the transcriptional landscape governed by YAP/TAZ and JAK/STAT, either generating signaling conflicts or reinforcing an inhibitory microenvironment depending on the pathological stage.

Ultimately, the convergence of these localized metabolic outposts and macro-level mechano-chemical cascades highlights that noncanonical Nogo-A signaling functions as a dual-layered coordinator. It simultaneously freezes local translational capacity at the micro-scale via mTORC1 restriction and manipulates macro-scale transcriptional homeostasis through RhoA-driven mechanical tension. Consequently, bypassing the extracellular inhibitory blocks requires integrative therapeutic strategies that simultaneously target both the metabolic and mechano-chemical dimensions of the downstream signaling network.

## Nogo-A Signaling Network and its Multi-target Regulatory Potential in Neuroplasticity and Neurodevelopmental Disorders

### Synaptic Plasticity: Rapid Modulation of Dendritic Spines and Synaptic Proteins

Current evidence suggests that neuroplasticity homeostasis emerges from a multifaceted regulatory network rather than a solitary dominant pathway. As early as 2016, Kellner’s team demonstrated that Nogo-A could remodel the F-actin cytoskeleton of dendritic spines via the ROCK/Cofilin axis within minutes, thereby directly modulating AMPA receptor insertion efficiency (Kellner et al. 2016). This rapid regulatory mechanism involves the Cofilin-mediated severing of actin filaments, which transiently prune synaptic structures to prevent excessive reinforcement. Building upon this, Zagrebelsky’s laboratory further dissected the NgR1 receptor complex in the Schaffer collateral-CA1 pathway. They demonstrated a distinct functional segregation where LINGO-1 predominantly restricts LTP, whereas p75^NTR^ specifically mediates LTD (Iobbi et al. 2017). When Nogo-A was inhibited, the Morris water maze test revealed a significant improvement in spatial learning and memory formation in mice (Zagrebelsky et al. 2017). This suggests that the restriction imposed by Nogo-A on learning and memory may involve latent mechanisms independent of cytoskeletal dynamics, such as translational reprogramming of proteins in the postsynaptic density region. With respect to age-related differences, findings from the postoperative neurocognitive dysfunction (pNCD) model remain more contentious. Nogo-66-NgR1 signaling activation in the mouse hippocampus induces F-actin depolymerization, decreases spine density, impairs AMPA receptor surface delivery, and suppresses excitatory synaptic transmission. NEP1-40 and Fasudil can reverse these effects and enhance cognitive performance, albeit exclusively in aged mice. In contrast, recovery in young mice appears to rely more heavily on endogenous miRNA regulation (Jia et al. 2025). In this context, some researchers propose that the “braking effect” of Nogo-A signaling in the aged brain is excessively amplified. Furthermore, many scholars suggest that microenvironmental inflammatory factors may synergistically potentiate this inhibitory mechanism (Doorduin et al. 2009; Dilmen et al. 2024; Borchers et al. 2023). This academic divergence underscores the intricate nature of the Nogo-A regulatory network, it functions as both a guardian of synaptic homeostasis and potentially transforms into a driver of cognitive decline under pathological conditions.

### Mechanism Underlying the Balance Between Axon Regeneration and Plasticity Limitation

The efficacy of Nogo-A blockade in promoting functional regeneration continues to be a long-standing focal point of investigation within the field of spinal cord injury. We conducted a preliminary summary and comparative analysis of various intervention methods and their effects across multiple experimental models (see Table 2). Through this comparison, we can gain deeper insights into the mechanisms of Nogo-A in neuroplasticity and disease intervention.”

Preclinical studies conducted by Yazdi’s group demonstrated that the anti-Nogo-A antibody enhanced neurite growth in PC-12 cells and promoted axonal regeneration across injury scars in a primate model (Yazdi et al. 2016). However, certain experiments have yielded less than ideal outcomes. In the chronic compression model, the same treatment restored only 40% of motor function. When translated to human clinical trials, although efficacy was observed, there was also an associated risk of abnormal synaptic connections (Powers et al. 2023). Researchers generally attribute this bottleneck to several factors: (1) The large molecular weight (~ 150 kDa) of anti-Nogo-A antibodies (e.g., ATI355) limits their ability to effectively penetrate the intact blood-brain barrier, leading to insufficient exposure of central nervous system targets (Rust et al. 2024). (2) Circulating anti-Nogo-A antibodies may bind LINGO-1 on peripheral T cells, triggering IFN-γ secretion, thereby exacerbating neuroinflammation and partially counteracting the central therapeutic effect (Foale et al. 2017). (3) Nogo-A exhibits cross-reactivity with non-target receptors such as integrin αv, potentially disrupting angiogenesis or synaptic homeostasis (Hu and Strittmatter 2008). Additionally, some studies suggest that these discrepancies might be linked to the pathological microenvironment. For instance, in Alzheimer’s disease models, anti-Nogo-A treatment restores LTP, but this requires dual support from NMDAR signaling and de novo protein synthesis (Pavon et al. 2023).

**Table 2:** Nogo-A intervention efficacy comparison in experimental models

Model	Intervention method	Outcome	Mechanism	Limitations
Spinal cord injury in mice	Anti-Nogo-A Antibody	Axon regeneration increased by 40%, partial recovery of motor function	Blockade of NgR1/RhoA pathway	Risk of aberrant connections due to indiscriminate plasticity opening; requires paired targeted rehabilitation to avoid maladaptive rewiring (Powers et al. 2023; Schwab and Strittmatter 2014)
APP/PS1 AD model	Delivery of miR-182-5p	Recovery of LTP, reduced tau phosphorylation	Downregulation of Nogo-A expression	Dose-dependent effects; significant hurdles in vivo delivery efficiency and off-target risks; years away from stable clinical tool (Soto et al. 2022)
Lizard retina injury	Activation of cAMP/EPAC	Axon regeneration capacity increased by 300%	Bypassing inhibition by RtN4 (Nogo homolog)	cAMP/EPAC pathway activity insufficient in mammals (Lang et al. 2017)
Drosophila neural circuit	Activation of Robo/Slit System	Maintenance of synaptic homeostasis (no Nogo homolog)	Alternative inhibitory mechanism	Compatibility with mammalian signaling unknown (Chen et al. 2020)

Subtly, the discovery of miR-182-5p provides new ideas for precise regulation. This miRNA can specifically silence Nogo-A expression, but its effect is dose-dependent: low doses promote axon branching, and high doses instead cause growth cone collapse (Soto et al. 2022). This seems to indicate that moderate disinhibition is the key to regeneration. Furthermore, this regulation may be achieved through epigenetic modifications, such as the dynamic interaction between histone deacetylase (HDAC) and Nogo-A promoter (D’Mello 2019). At the same time, if miR-182-5p is encapsulated by liposomes or exosomes, it can not only cross the blood-brain barrier, but also reduce the risk of off-target by sequence-specific silencing of Nogo-A mRNA. However, there is no direct evidence to support this hypothesis, and subsequent studies are urgently needed to verify it by single-cell chromatin accessibility analysis.

### Neurovascular Interactions and Permeability of the Blood-Brain Barrier

The role of Nogo-A in the vasculature is significantly more intricate than previously appreciated. Tang’s team demonstrated that Nogo-A knockout mice exhibited a increase in cerebrovascular branch points; however, the associated neovascularization displayed a tendency toward leakage (Wälchli et al. 2017). This “paradox of quantity and quality” highlights its dual functionality. In the midbrain infarction model, the RTN4/S1PR2 complex activates autophagy via BECN1 phosphorylation, whereas inhibition of this pathway promotes angiogenesis but exacerbates blood-brain barrier disruption (Tang et al. 2020; Xiao et al. 2022). In this context, Thomas posits that Nogo-A may function as a “pressure sensor” for vascular homeostasis, restricting excessive proliferation to prevent hemorrhage during the early ischemic phase while requiring timely withdrawal to facilitate repair in later stages (Wälchli et al. 2013).

The latest breakthrough originates from extracellular vesicles (EVs) research: neuronal-derived EV Nogo-A not only inhibits fibroblast migration via S1PR2 but also crosses the BBB to enter the peripheral circulation (Rust et al. 2024). This elucidates why serum Nogo-A levels are positively correlated with the extent of brain damage in stroke patients. It remains to be determined whether these extracellular vesicles (EVs) function as proactive “distress signals” or passive “death markers” of injury. Furthermore, EV-associated Nogo-A is hypothesized to indirectly modulate neuroinflammation by co-opting the chemotactic recruitment of peripheral immune cells (Guo et al. 2020; Marostica et al. 2020). These hypotheses have yet to be validated through in vivo imaging and necessitate further experimental investigation. Additionally, the heterogeneity of EVs (e.g., differences between exosomes and microvesicles) may complicate the interpretation of conclusions.

### Nogo-A’s Role in Neuroinflammation, Oxidative Stress, and its Association with Disease Progression

**Fig. 2:**
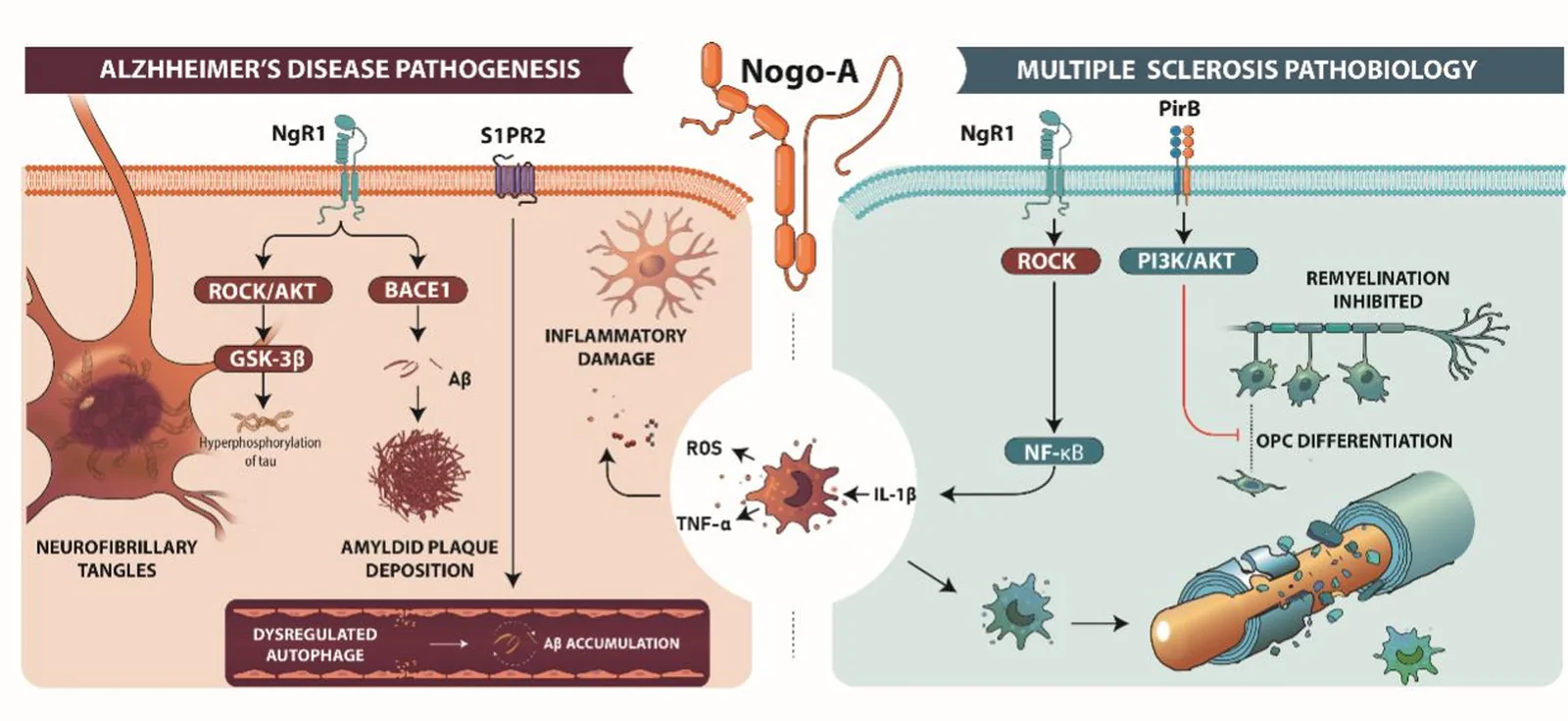
Mechanistic Insights into the Nogo-A Signaling Network Across Disease Pathologies. Left Panel: Alzheimer’s Disease (AD) pathogenesis. Nogo-A interacts with NgR1 to activate ROCK/AKT and GSK-3β, leading to tau hyperphosphorylation and neurofibrillary tangles. Simultaneously, NgR1 and S1PR2 signaling upregulates BACE1 and dysregulates autophagy, driving Aβ accumulation and amyloid plaque deposition. These pathways converge to induce inflammatory damage via ROS and cytokine (TNF-α, IL-1β) release. Right Panel: Multiple Sclerosis (MS) pathobiology. Nogo-A engages PirB and NgR1 on oligodendrocyte precursor cells (OPCs) and microglia. PirB-mediated PI3K/AKT suppression inhibits OPC differentiation and remyelination. Concurrently, NgR1-mediated ROCK/NF-κB activation promotes microglial secretion of IL-1β, further exacerbating inflammatory damage and demyelination

Significant controversy persists regarding the mechanistic involvement of Nogo-A in Alzheimer’s disease, with researchers divided on whether the protein actively contributes to or is merely associated with neurodegenerative progression. Studies have demonstrated that the Nogo-66/NgR1 axis promotes Aβ production by activating BACE1, a process that can be blocked by Fasudil (Xie et al. 2021; Guo et al. 2020; Yang et al. 2020). Fei Xiao et al. further revealed that Nogo-A enhances tau vulnerability via ROCK/AKT/GSK3β signaling, leading to increased tau hyperphosphorylation, synaptic loss, and cognitive decline. The knockdown of Nogo-A significantly slowed disease progression in AD mouse models (Wang et al. 2024). Conversely, other researchers argue that the pro-apoptotic effects mediated by p75^NTR^ may represent the central mechanism. In AD brains, p75^NTR^ expression is upregulated, interacting with amyloid β-peptide to activate pro-apoptotic signaling pathways. Recent clinical trials have shown that LM11A-31, a p75^NTR^ antagonist, effectively reduces CSF tau levels and improves cognitive function in patients with mild-to-moderate AD (Shanks et al. 2024). Furthermore, preclinical studies in AD mouse models have demonstrated that p75^NTR^ antagonists can effectively delay tau phosphorylation and inhibit associated pathologies (Yang et al. 2020).Nogo-A This difference can be attributed to the redundancy within the molecular network, where distinct receptors may assume dominance at various stages of disease progression. Moreover, Nogo-A/NgR1 signaling intersects with TLR4, NF-κB, and NLRP3 inflammatory pathways, playing a role in the modulation of neuroinflammation in multiple sclerosis, Parkinson’s disease, schizophrenia, and other disorders (Tondo et al. 2019; Nheu et al. 2022). Research on tightly coupled synchronous synapse-nuclear signal transduction suggests that Nogo-A might influence the activation of CREB and other nuclear transcription factors, thereby mediating the imbalance of synapse-nuclear communication and exacerbating cognitive impairment (Nheu et al. 2022). In the postoperative cerebral ischemia-reperfusion (CI/RI) model, moxibustion intervention was shown to downregulate the expression of Nogo-A, NgR1, p75^NTR^, and LINGO-1, improving neurological function and reducing cerebral infarction volume, which further underscores the multifaceted roles of Nogo-A in neuroinflammation and ischemic injury (Min et al. 2023). Figure 2 illustrates the role of the Nogo-A receptor in Alzheimer’s disease and multiple sclerosis.

In the context of oxidative stress, overexpression of Nogo-A in H₂O₂-treated oligodendrocytes was found to elevate ROS levels (Wang et al. 2021b). This observation implies that Nogo-A might contribute to the establishment of oxidative stress memory via epigenetic modifications, such as DNA methylation. Data from the plateau hypoxia model demonstrated that Nogo-A knockout could restore mitochondrial membrane potential; however, this effect was contingent upon the synergistic activation of HIF-1α and PGC-1α (Fang et al. 2025). Such a multi-factorial coupling mechanism may elucidate the repeated failures of simplistic antioxidant therapies in clinical trials.

Finally, the identification of RACK1 offers a novel perspective on cross-pathway regulation. This scaffold protein not only modulates NMDA receptor function but also influences APP cleavage, indicating its potential role as a “molecular bridge” connecting the Nogo-A and inflammatory pathways (He et al. 2024). Nevertheless, no study to date has directly elucidated the physical interaction between RACK1 and NgR1, thereby highlighting a critical knowledge gap that warrants further investigation.

## A Unified Conceptual Framework: From Spatiotemporal Mechanisms to Disease Progression

The complexity of Nogo-A signaling arises from its ability to engage different receptors depending on the cellular context and the stage of pathology. To move beyond a “list-like” description, here we propose a conceptual framework to understand the spatiotemporal dynamics and cell-type specificity of Nogo-A interactions. Note that this model remains hypothetical and requires further in vivo validation.

### Spatiotemporal Dynamics: From Acute Injury to Chronic Degeneration

The preference for specific Nogo-A signaling pathways shifts significantly over the course of a pathological event, such as an ischemic stroke(Rust et al. 2024).

Acute Phase (0–24 h post-injury): In the immediate aftermath of injury, Nogo-A primarily signals through S1PR2 on both neurons and endothelial cells(Kempf et al. 2014). This interaction triggers rapid RhoA activation, leading to acute growth cone collapse in neurons and increased vascular permeability in the blood-brain barrier (BBB) (Tang et al. 2020). This phase is characterized by a “defense-first” response, where Nogo-A limits further damage by restricting structural fluctuations, albeit at the cost of initial regenerative potential (Tang et al. 2020; Rust et al. 2019).

Subacute to Chronic Phase (7d+ post-injury): As the lesion stabilizes, the signaling weight shifts toward NgR1 and PirB (Akbik et al. 2013; Raiker et al. 2010). These receptors mediate the long-term restriction of axonal sprouting and synaptic plasticity, effectively “closing” the window of heightened plasticity (Akbik et al. 2013). In this stage, Nogo-A acts as a stabilizer of the remaining neural circuits, preventing aberrant connections but also hindering functional recovery(Schwab and Strittmatter 2014).

### Differences in Hippocampal Subregions and the Inhibition of CA2 Plasticity

The hippocampus is characterized by significant functional and molecular differentiation across its longitudinal and transverse axes (Strange et al. 2014). The molecular mechanisms underlying the heightened sensitivity of synaptic plasticity in the hippocampal CA2 region to Nogo-A signaling warrant further investigation. In neurodegenerative contexts, rather than acting as an absolute primary driver of synaptic loss, Nogo-A upregulation is likely a pathological consequence that secondarily exacerbates the condition by further suppressing compensatory plasticity. Furthermore, it has been demonstrated that Thrombospondin-1 secreted by astrocytes may disrupt NgR1 signaling via integrin α2β1, highlighting the regulatory role of non-neuronal elements in regional specificity (Wang et al. 2024).

The age factor contributes to the complexity of recovery outcomes. Following ischemic stroke in both pediatric and adult mice across different ages, synaptic plasticity damage mediated by Nogo-A receptor signaling is more readily reversible in younger mice. Inhibition of the Nogo-A receptor accelerates synaptic repair; however, adult mice exhibit weaker reliance on this pathway (Orfila et al. 2020). This phenomenon can be attributed to “developmental signaling compensation,” wherein stage-specific compensatory mechanisms synergistically act during development. For instance, the robust activity of the BDNF/TrkB pathway in young brains may partially counteract the inhibitory effects of Nogo-A (Vanacore et al. 2024). Additionally, mitochondria in young neurons maintain synaptic stability via MfN2-dependent calcium buffering mechanisms (Zaman and Shutt 2022).

### Modulation of Nogo-A/NgR1 Signaling in Multiple Sclerosis and Microglia

“Microglia exhibit a dual role in neural repair”, as prominently demonstrated in multiple sclerosis (MS) research. Petratos’s team revealed that activated microglia in lesion areas maintain the M1 phenotype via NgR1 signaling, with their secretion of IL-1β directly inhibiting oligodendrocyte precursor cell (OPC) differentiation (Nheu et al. 2022) (Fig. 2). Blocking this pathway facilitates the transition of microglia toward an anti-inflammatory M2-like phenotype, evidenced by reduced secretion of pro-inflammatory cytokines such as IL-1β and TNF-α. Simultaneously, upregulation of M2 markers like Arg1 and CD206 was observed, leading to enhanced myelin debris clearance and remyelination, thereby improving functional deficits. However, recent studies indicate that M1-like microglia are not a homogeneous population; approximately 30% of these cells co-express AMIGO3 and Arg1, exhibiting mixed pro-phagocytic/anti-inflammatory characteristics (Stratoulias et al. 2023). Whether this “heterogeneous state” is regulated by Nogo-A remains unresolved by current research. Treatment strategies remain controversial, as anti-Nogo-A antibodies significantly promoted remyelination in a primate MS model but yielded limited efficacy in human clinical trials, improving motor function while demonstrating weaker effects on cognitive recovery (Mohammed et al. 2020). In this context, Kartje posited that the inefficiency of BBB penetration represents a critical bottleneck. Furthermore, a more speculative hypothesis suggests that LINGO-1-mediated trans-activation of peripheral T cells might antagonize the central therapeutic effects (Jepson et al. 2012; Foale et al. 2017). Local delivery of miR-182-5p exhibits distinct advantages: it not only downregulates Nogo-A but also cleaves the mRNA of the pro-inflammatory factor TNF-α induces the targeted degradation of the mRNA encoding, thereby achieving dual-pathway inhibition (Soto et al. 2022). This RNA-based therapeutic approach evokes comparisons to the precision editing capabilities of the CRISPR-Cas13d system, albeit with the lingering concern of off-target effects acting as an ever-present risk.

### The Dynamic Expression of Nogo-A During Puberty and Aging is Associated with the Development of Diseases

The adage “The brain remodels in adolescence and solidifies in old age” finds molecular support in the dynamics of Nogo-A signaling. From birth through adulthood and into old age, the transcriptional levels of Nogo-A and its 11 key ligands, receptors, and regulatory molecules exhibit gene- and region-specific dynamic changes across various brain regions. The expression differences in the cerebral cortex, hippocampal CA regions, and amygdala subregions are particularly significant, providing a foundation for the plasticity states characteristic of different ages (Smedfors et al. 2018). Analogous to the cross-tissue spatiotemporal regulation pattern of signaling pathways observed in the “dauer” program of Caenorhabditis elegans, the Nogo-A signaling pathway makes adaptive decisions at distinct developmental stages to balance structural plasticity with functional stability (Godoy and Hochbaum 2023). In comparison to conditions such as multiple sclerosis and AD, the adolescent brain exhibits greater plasticity, with relatively low expression of the Nogo-A/NgR1 signal, which facilitates neural circuit growth and learning. With aging, this signal becomes progressively upregulated, closely correlating with cognitive decline and synaptic loss (Jia et al. 2025; Nibuya et al. 2024).

From an evolutionary perspective, certain non-mammalian species, such as the Gallotia lizard, exhibit a significantly greater capacity for regenerating retinal ganglion cell (RGC) axons compared to mammals, despite similar expression levels of the Nogo-A homolog RtN4. The discrepancy in retinal ganglion cell (RGC) axonal regeneration between non-mammalian species, such as the *Gallotia* lizard, and mammals—despite comparable levels of the Nogo-A homolog RtN4—may be attributed to differential downstream signaling. Specifically, the activation of the cAMP/PKA signaling pathway is hypothesized to neutralize the inhibitory function of RTN4, representing a potential biomimetic strategy for human therapeutic intervention (Lang et al. 2017). The evolutionary loss of this robust regenerative mechanism in mammals has been theorized to be a necessary biological trade-off for the enhanced synaptic stability required by the highly complex mammalian central nervous system (Schwab and Strittmatter 2014). Nevertheless, studies on Drosophila mutants have demonstrated that even in the absence of Nogo homologs, neural circuits can maintain homeostasis through the Robo/Slit system (Correa et al. 2023), implying that Nogo-A signaling may represent just one of several plasticity “brake systems”.

## Clinical Translation of Nogo-A-Targeted Therapies: Navigating Challenges and Operational Strategies

**Fig. 3:**
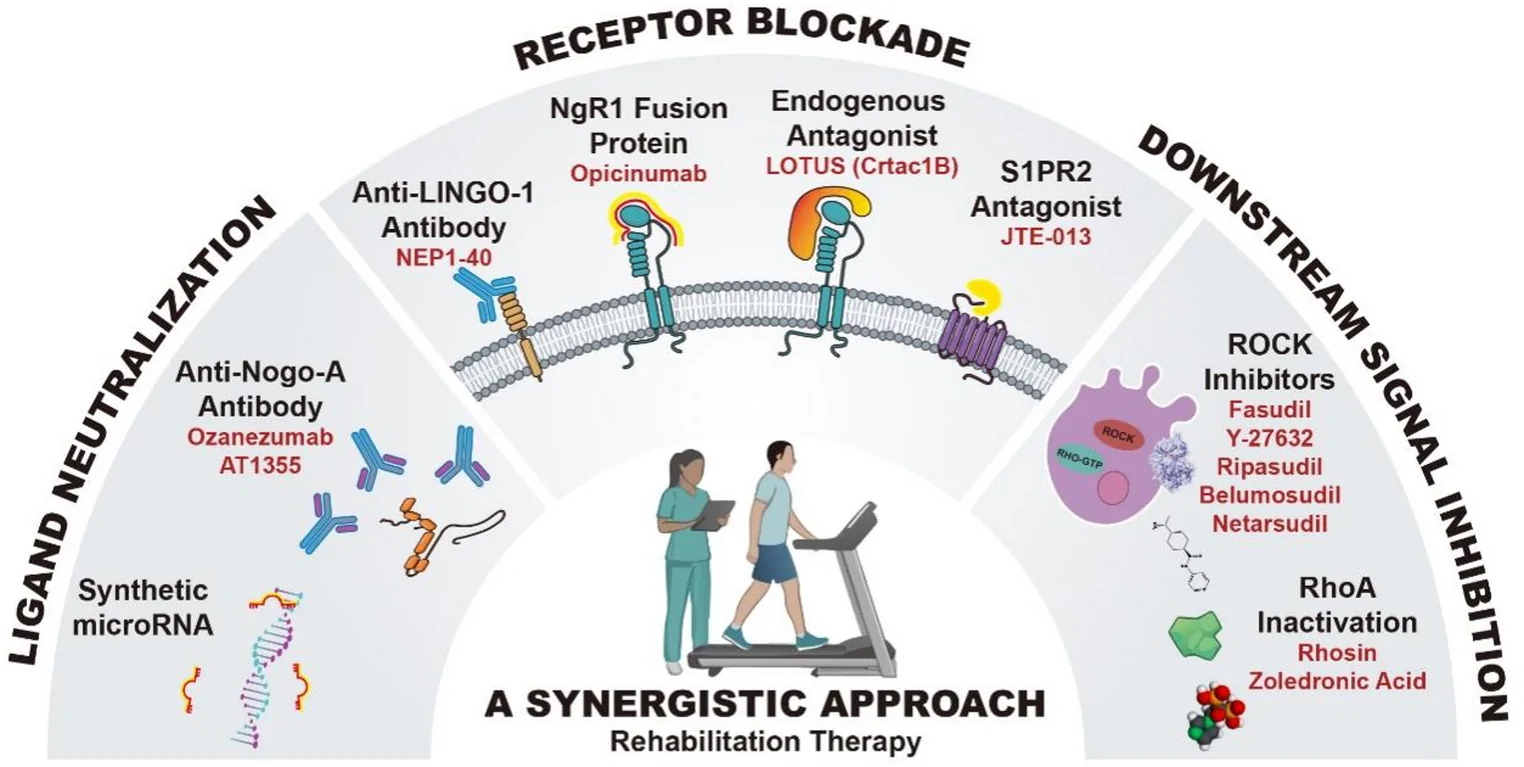
Therapeutic modalities and clinical translation strategies targeting the Nogo-A signaling network. The schematic categorizes pharmacological interventions into three primary strategies: (1) Ligand neutralization, including anti-Nogo-A antibodies (e.g., Ozanezumab, AT1355) and synthetic microRNA; (2) Receptor blockade, using antibodies, fusion proteins, and antagonists against LINGO-1 (NEP1-40), NgR1 (Opicinumab), and S1PR2 (JTE-013), as well as leveraging potent endogenous regulators such as LOTUS (Crtac1B) to intrinsically antagonize NgR1 and PirB signaling complexes; and (3) Downstream signal inhibition, utilizing ROCK inhibitors (e.g., Fasudil, Netarsudil) and RhoA inactivators (e.g., Rhosin, Zoledronic Acid). These molecular interventions are depicted alongside physical rehabilitation therapy to highlight a synergistic approach for functional recovery

The translation of Nogo-A-targeted therapies from preclinical promise to clinical efficacy has encountered substantial translational hurdles. While emerging “beyond NgR” strategies that engage non-canonical receptors such as S1PR2 or PirB hold potential, they must first overcome profound pharmacological and physiological barriers, including the need for a nuanced understanding of their pleiotropic systemic functions and limited CNS accessibility (Correa et al. 2023; Rust et al. 2019). As summarized in Fig. 3, the current therapeutic landscape targeting Nogo-A is actively exploring multiple strategic avenues to address these limitations. A primary operational focus is circumventing the restrictive BBB (Correa et al. 2023). Key delivery strategies under investigation include: (1) Nanocarrier Systems, utilizing brain-targeted exosomes or liposomes to encapsulate and deliver small-molecule inhibitors or regulatory miRNAs (e.g., miR-182-5p) (Tang et al. 2023); (2) Direct Intrathecal/Intracerebroventricular Administration, as employed in ongoing anti-Nogo-A antibody trials (e.g., ATI355) (Kucher et al. 2018), which bypasses the BBB entirely albeit via an invasive route; and (3) Focused Ultrasound (FUS), a technique to achieve transient, localized BBB opening to facilitate the entry of high-molecular-weight therapeutic antibodies. Collectively, these innovative delivery platforms represent critical steps toward realizing the clinical potential of Nogo-A pathway modulation. Beyond CNS delivery hurdles, safety and specificity bottlenecks represent critical limitations in clinical reality. The risk of abnormal connections during anti-Nogo-A treatment remains a significant concern; anti-Nogo-A treatments may induce indiscriminate opening of critical plasticity windows. Without targeted rehabilitation, this ‘excessive plasticity at the wrong time’ can lead to maladaptive rewiring, such as neuropathic pain or spasticity (Powers et al. 2023; Schwab and Strittmatter 2014). Furthermore, regarding novel genetic approaches, while promising conceptually, miRNA-based therapies currently face significant hurdles regarding in vivo delivery efficiency and off-target effects, meaning they are still years away from becoming stable clinical tools (Soto et al. 2022).

In addition to these exogenous interventions, endogenous modulators play a crucial role in shaping the translational landscape. Specifically, lateral olfactory tract usher substance (LOTUS, also known as Crtac1B) functions as a highly potent endogenous antagonist of Nogo-A signaling. As highlighted by Schwab and Strittmatter (2014) and established by comprehensive in vitro and in vivo studies, LOTUS directly binds to NgR1 and PirB, effectively blocking their pathological interaction with all four types of myelin-associated inhibitory ligands, including Nogo-A, MAG, OMgp, and BLyS. Its soluble form can interfere with the NgR1-p75^NTR^ coreceptor complex. These combined actions completely suppress downstream axonal growth inhibition and promote functional neuroregeneration (Schwab and Strittmatter 2014; Sato et al. 2011; Kurihara et al. 2014, 2020; Kawakami et al. 2018).

Furthermore, establishing a clear strategic framework requires integrating these exogenous and endogenous axes into a multi-layered combinatorial approach. Given that convergent platforms like NgR1 and PirB bind multiple inhibitory ligands beyond Nogo-A, targeting Nogo-A alone may be pharmacologically insufficient to fully disinhibit downstream signaling. Future translational directions must therefore emphasize combinatorial strategies: combining ligand-level neutralization (such as anti-Nogo-A antibodies) with endogenous receptor modulation (such as LOTUS upregulation) and downstream enzymatic blockade (such as RhoA/ROCK inhibitors). Synchronizing this molecular cocktail with the aforementioned targeted physical rehabilitation represents a comprehensive therapeutic paradigm to maximize neuroplasticity while avoiding maladaptive rewiring. Figure 3 illustrates therapeutic modalities and clinical translation strategies targeting the Nogo-A signaling network.

## Conclusions and Future Prospects

“Future neural repair strategies may need to move beyond reliance on a single analytic targeting approach.” This assertion becomes increasingly compelling when considering the intricate complexity of the Nogo-A signaling network. For instance, intranasal administration of anti-Nogo-A antibodies has been shown to significantly enhance functional recovery in whole-brain distribution within a mouse stroke model. However, clinical trials have highlighted a critical bottleneck regarding the penetration efficiency across the hemorrhagic brain barrier (Correa et al. 2023). This “gap between ideal and reality” aptly encapsulates the essence of the Nogo-A signaling network: it is neither a straightforward inhibitory switch nor an isolated axis of action, but rather a sophisticated functional integration network encompassing transmembrane receptors, intracellular kinases, and even epigenetic regulation mechanisms. From a molecular mechanism perspective, the interaction of Nogo-A with receptors such as NgR1 and PirB functions akin to a precise multifunctional regulator, capable not only of modulating the metabolic throttle of PI3K/Akt/mTOR (Bi and Quan 2018; Seiler and Widmer 2015) but also initiating the cytoskeletal brake mediated by RhoA/ROCK (Liu et al. 2021; Chang et al. 2016). Moreover, this regulatory process exhibits spatiotemporal specificity, exemplified by observations in the hippocampus of AD mice where the colocalization regions of Nogo-A with p75^NTR^ closely align with tau phosphorylation hotspots (Sui et al. 2015), suggesting that localized interventions might hold greater therapeutic potential compared to global inhibition. In addition, it is noteworthy that deconstructing the Nogo-A signaling network reveals its dual role in neuroinflammation. On one hand, Nogo-A exerts a protective inhibitory function by restricting excessive inflammatory responses, such as inhibiting microglial M1 phenotype activation, thereby maintaining neural homeostasis (Tondo et al. 2019; Nheu et al. 2022). On the other hand, aberrant interactions between Nogo-A and its receptors, such as NgR1 and PirB, can amplify the release of pro-inflammatory factors (e.g., IL-1β and TNF-α) and oxidative stress, contributing to the progression of neurodegenerative diseases (Wang et al. 2024; Fang et al. 2025). This duality underscores the clinical translational challenge of targeting the Nogo-A network: distinguishing precisely between its protective and pathological signaling pathways is essential to prevent decompensation caused by global inhibition. For instance, in AD, while anti-Nogo-A antibodies enhance synaptic plasticity, they may also exacerbate peripheral inflammation by disrupting the physiological immunomodulatory role of Nogo-A (Freund, 2007).

Future breakthroughs may emerge from unexpected interdisciplinary intersections. We could attempt to integrate multi-omics data related to Nogo-A for comprehensive data preprocessing, including cleaning, standardization, and feature extraction. A preliminary Nogo-A network was constructed based on existing biological knowledge and further visualized and analyzed. Additionally, the implications of insect nervous systems should not be overlooked: Drosophila, which lacks a Nogo homologue, maintains loop homeostasis via the Robo/Slit system (Correa et al. 2023), an alternative evolutionary strategy that implies mammals might possess an undiscovered “backup brake.” While optimism is warranted, it must be balanced with caution. For example, the convenience of nasal delivery is promising, yet whether antibodies can effectively penetrate brain regions beyond the olfactory bulb remains uncertain. Epigenetic editing technology holds potential for reversing methylation of the Nogo-A promoter; however, off-target effects could potentially activate proto-oncogenes. This process resembles repairing an intricate evolutionary mechanism, rusty gears must be cleaned without disrupting the delicate clockwork.

In summary, the deconstruction of the Nogo-A signaling network is fundamentally reshaping our understanding of neural repair. As the dynamic regulatory mechanisms of the Nogo-A signaling network continue to be unveiled at molecular, cellular, and systemic levels, differential intervention strategies leveraging multi-target collaboration are anticipated to yield revolutionary breakthroughs in neural repair and the treatment of neurodevelopmental disorders.

## Data Availability

No datasets were generated or analysed during the current study.
